# Supplementary material for: The prognostic value of gait speed in hemodialysis patients: A prospective observational study
Source: PLoS One. 2026 Mar 18;21(3):e0343612. doi: 10.1371/journal.pone.0343612 (PMC12998855; doi:10.1371/journal.pone.0343612)
Supplement: S1 File — (PDF) [file pone.0343612.s001.pdf]

S1. Methods used for biochemical measurements.

Biochemist Variables	Method
Vit D (ng/ml)	Chemiluminescence
PTH (pg/ml)	Chemiluminescence
C-PR (pg/ml)	Turbidimetry
Kt/v	Urea Kinetic Modeling
Hemoglobin (g/dL)	Automated counting by flow cytometry
Hematocrit (%)	Automated counting by flow cytometry
Albumin (g/dL)	Colorimetric
Globulin (g/dL)	Immunonephelometry
Potassium (g/dL)	Ion-selective electrode
Sodium (g/dL)	Ion-selective electrode
Phosphorus (g/dL)	Colorimetric
Calcium (mg/dL)	Colorimetric
LDL (mg/dL)	Enzymatic colorimetric
HDL (mg/dL)	Enzymatic colorimetric
Triglycerides (mg/dL)	Enzymatic colorimetric
Ferritin (ng/dL)	Chemiluminescence
Iron (µg/dL)	Colorimetric
Alkaline phosphatase (U/L)	Colorimetric

Legend: Kt/v fractional urea clearance, PTH parathormone, C-RP C-reactive protein, LDL low-density lipoprotein, HDL high-density lipoprotein.
