# Supplementary material for: The prognostic value of gait speed in hemodialysis patients: A prospective observational study
Source: PLoS One. 2026 Mar 18;21(3):e0343612. doi: 10.1371/journal.pone.0343612 (PMC12998855; doi:10.1371/journal.pone.0343612)
Supplement: S2 File — (PDF) [file pone.0343612.s002.pdf]

Sex	Age	Diabetes	Dry_weigh	Fat	BMI	ALM	Gait_speec	HDvintage_
2	61,00	1,00	55,20				1,09	151,00
2	69,00	0,00	95,30		24		0,77	804,00
2	47,00	1,00	55,00	12,90	20	19,23	1,06	2249,00
2	66,00	0,00	56,30	27,60	23	15,96	0,86	3487,00
1	55,00	0,00	42,90	30,70	20	10,75	1,00	312,00
2	39,00	0,00	48,60	14,60	20	16,04	1,01	2186,00
2	78,00	0,00	79,70	15,70	27	26,46	0,95	67,00
2	71,00	0,00	61,10	18,90	22	19,84	1,08	590,00
2	79,00	0,00	61,50				0,63	3090,00
2	67,00	1,00	58,80	19,70	23	18,16	0,94	660,00
1	66,00	0,00	53,10	37,50	22	12,83	0,61	1438,00
1	56,00	0,00	79,20	44,30	33	17,21	0,76	1430,00
1	45,00	0,00	51,10	35,20	21	14,27	0,94	826,00
1	55,00	0,00	41,90	25,00	18	11,70	0,74	1742,00
1	63,00	1,00	82,00	51,60	38	14,49	0,48	1920,00
2	48,00	1,00	68,20	33,90	29	19,40	0,93	397,00
1	52,30	0,00	66,60	45,90	27	34,41	0,93	65,00
1	48,60	0,00	50,10	29,30	19	33,65	1,18	254,00
1	61,60	0,00	39,00	40,20	23	21,96	1,18	133,00
1	68,30	0,00	54,50	32,00	25	35,61	1,28	2527,00
2	58,00	1,00	55,30	36,80	21	32,95	0,68	668,00
2	60,10	0,00	70,70	36,90	25	42,60	1,13	6094,00
2	58,50	0,00	67,30	28,90	23	45,44	1,14	2038,00
2	55,30	1,00	82,20	33,50	25	52,20	0,97	3675,00
1	63,70	0,00	59,90	43,90	28	32,20	1,07	2563,00
1	63,90	1,00	65,00	42,70	33	36,12	0,73	1743,00
2	79,00	1,00	69,80	42,60	30	17,32	0,78	1332,00
2	63,00	0,00	85,60	44,40	33	19,27	0,80	211,00
2	57,00	0,00	53,20	10,90	17	31,19	1,03	102,00
2	76,40	1,00	87,80	43,00	35	47,89	0,52	1258,00
1	56,00	0,00	65,10	41,90	28	35,90	1,03	135,00
2	53,90	1,00	65,70	26,60	26	45,75	0,60	323,00
1	51,00	1,00	53,30	39,70	21	30,59	0,92	1876,00
2	32,10	1,00	52,90	9,40	20	45,92	1,15	40,00
1	27,00	0,00	44,80	38,10	19	10,57	1,20	871,00
1	27,00	0,00	46,10	26,40	20	12,55	1,54	1696,00
2	30,00	1,00	100,10	33,20	33	33,92	1,38	609,00
1	50,00	0,00	68,20	40,10	28	17,21	0,86	142,00
2	47,00	0,00	71,50	18,40	24	25,87	1,35	456,00
2	34,00	0,00	48,10	22,50	19	13,99	1,34	11,00
2	59,00	1,00	94,70	33,50	29	28,92	1,18	408,00
1	61,00	1,00	85,20	37,90	38	19,52	1,37	452,00
1	20,00	0,00	49,90	32,00	20	11,91	1,04	241,00
1	41,00	0,00	58,70	45,40	24	12,89	1,28	447,00
1	26,00	0,00	57,20	38,10	23	14,43	1,29	1048,00
2	30,00	0,00	59,00	15,20	23	20,01	1,09	368,00
2	23,00	0,00	56,60	19,90	20	19,05	0,72	2247,00
2	52,00	0,00	64,90	28,80	23	18,76	1,54	56,00
2	45,00	0,00	76,50	31,20	28	24,68	1,46	2949,00

2	59,00	0,00	61,20	32,80	24	17,63	0,97	297,00
2	52,00	0,00	69,80	37,60	26	18,49	1,16	695,00
2	59,00	0,00	58,60	17,70	21	19,44	1,14	3483,00
2	49,00	0,00	54,70	20,30	20	17,28	1,13	1760,00
2	52,00	0,00	66,80	26,40	23	20,95	1,21	46,00
2	59,00	0,00	79,30	33,10	30	24,29	1,24	612,00
2	78,00	1,00	74,60	45,10	30	16,46	0,72	581,00
2	48,00	1,00	79,90	20,90	26	21,84	1,00	302,00
2	27,00	0,00	64,70	11,70	22	24,22	1,28	158,00
2	29,00	0,00	56,50	12,80	20	20,08	1,21	2524,00
2	57,00	0,00	60,20	18,90	24	20,88	0,99	2524,00
1	55,00	0,00	57,90	43,00	28	12,45	0,81	588,00
2	62,00	0,00	69,00	25,70	27	20,89	1,14	1495,00
2	52,00	0,00	63,00	17,30	28	21,72	1,18	-33,00
2	79,00	0,00	57,00	36,60	22	14,54	0,89	696,00
1	65,00	1,00	67,60	43,80	34	15,19	0,78	1983,00
1	39,00	0,00	73,60	43,40	31	17,38	1,43	449,00
2	39,00	0,00	66,00	24,00	21	20,73	1,72	687,00
1	29,00	1,00	50,90				1,30	707,00
2	35,00	0,00	50,20	17,60	19	17,32	1,28	744,00
1	29,00	0,00	39,10	15,60	19	12,06	1,36	4986,00
2	26,00	0,00					1,38	1295,00
2	40,00	0,00	44,50	13,10	19	15,69	1,71	1411,00
1	34,40	0,00	43,10	30,80	18	28,75	1,36	566,00
1	41,50	0,00	54,70	30,70	24	35,80	1,17	484,00
1	22,00	0,00	44,60	27,50	18	30,37	1,28	1178,00
2	26,90	0,00	58,50	23,90	22	41,71	1,74	569,00
2	45,90	0,00	83,40	34,80	29	51,80	1,14	126,00
1	41,90	0,00	75,20	47,70	31	37,68	1,35	773,00
2	59,30	1,00	63,50	26,30	24	44,69	1,59	442,00
2	28,20	0,00	68,40	14,00	22	54,80	1,46	1165,00
2	45,70	0,00	94,30	35,10	32	58,56	1,25	210,00
2	41,60	1,00	50,90	14,10	17	41,64	1,61	121,00
1	61,70	0,00	49,10	36,20	21	29,66	0,98	135,00
2	69,70	0,00	57,50	16,30	23	45,39	1,24	1290,00
1	44,40	1,00	37,00	17,30	15	29,09	0,74	203,00
1	56,30	0,00	40,60	16,20	18	32,89	1,01	9539,00
2	43,00	0,00	79,50	23,20	27	58,47	1,46	8059,00
2	40,50	1,00	88,00	16,10	26	70,16	1,69	958,00
2	73,40	1,00	97,40	36,40	33	59,24	1,17	253,00
2	42,80	0,00	56,90	17,00	21	43,91	1,68	400,00
2	38,10	0,00	86,50	24,90	26	62,26	1,51	972,00
2	36,80	0,00	67,20	26,40	22	46,69	1,27	478,00
2	63,70	0,00	83,60	38,50	31	48,59	1,26	300,00
2	60,90	0,00	83,00	30,10	26	54,68	1,23	644,00
2	57,10	1,00	70,60	28,70	27	48,04	1,45	1093,00
2	28,00	0,00					1,41	3378,00
2	79,70	1,00	58,80	27,80	24	39,33	0,62	450,00
2	51,50	0,00	65,50	33,00	24	41,11	1,49	643,00
2	55,50	0,00	54,80	7,70	23	48,48	1,29	632,00

1	77,70	1,00	38,50	34,60	19	23,92	0,78	119,00
1	65,60	0,00	57,30	51,50	27	26,57	0,45	728,00
1	47,00	0,00	46,10	34,70	21	28,82	1,21	547,00
1	58,10	1,00	75,30	36,00	30	47,78	1,08	329,00
1	62,70	0,00	54,30	29,90	22	36,75	1,48	960,00
1	46,80	0,00	42,80	34,10	18	26,73	1,04	1002,00
2	72,60	1,00	100,40	42,40	35	54,82	1,19	660,00
1	47,00	0,00					1,31	73,00
1	59,30	1,00	50,70	41,80	22	28,27	1,64	746,00
2	49,40	0,00	75,30	32,90	29	48,25	1,29	742,00
2	32,20	0,00	55,50	23,90	21	40,53	1,22	521,00
1	68,50	0,00	48,50	38,70	20	28,27	1,48	19,00
1	42,10	0,00	94,80	49,50	36	45,97	1,37	420,00
2	78,30	0,00	61,30	30,90	22	39,82	1,03	1400,00
2	20,70	0,00	44,50	23,70	17	32,09	1,18	1264,00
2	52,10	0,00	69,10	40,60	26	39,48	1,18	489,00
1	38,10	0,00	50,00	32,60	21	31,80	1,35	80,00
2	56,20	0,00	65,00	31,30	25	44,60	1,28	4799,00
2	80,00	0,00					0,92	110,00
2	35,50	0,00	71,10	32,70	26	45,79	1,37	1322,00
2	69,30	1,00	91,00	38,90	32	52,42	0,73	89,00

HDvintage_	followup_c	followup_r	Mortality	VitD	PTHpgml	R_PCmlL	ktv	hb
5,03	273,00	9,10	1,00	27	256	6	1,41	9,20
26,80	264,00	8,80	1,00	46	201	3	1,11	10,30
74,97	1120,00	37,33	1,00	26	807	4	1,40	9,40
116,23	714,00	23,80	1,00	73	509	16	1,56	11,30
10,40	979,00	32,63	1,00	47	999	24	1,21	10,40
72,87	705,00	23,50	1,00	39	434	11	1,63	12,40
2,23	528,00	17,60	1,00	23	382	4	1,13	11,90
19,67	528,00	17,60	1,00	44	321	3	1,30	12,30
103,00	757,00	25,23	1,00	50	494	2	1,58	11,50
22,00	915,00	30,50	1,00	22	186	6	1,22	11,40
47,93	1010,00	33,67	1,00	20	337	2	1,81	11,50
47,67	1150,00	38,33	1,00	15	343	44	1,51	10,90
27,53	542,00	18,07	1,00	28	628	5	0,98	10,50
58,07	139,00	4,63	1,00	18	130	18	1,54	10,00
64,00	570,00	19,00	1,00	16	655	3	1,58	12,70
13,23	321,00	10,70	1,00	19	135	2	1,01	9,80
2,17	250,00	8,33	1,00	35	256		1,69	9,80
8,47	225,00	7,50	1,00	28	396	64	1,92	9,00
4,43	139,00	4,63	1,00	27	24	35	1,82	8,00
84,23	438,00	14,60	1,00	34	60	51	1,46	6,90
22,27	263,00	8,77	1,00	30	24	54	1,67	7,90
203,13	295,00	9,83	1,00	36	259	38	1,34	10,50
67,93	209,00	6,97	1,00	49	556	54	0,96	10,60
122,50	649,00	21,63	1,00	15	300	81	1,38	12,50
85,43	628,00	20,93	1,00	44	1290	60	1,61	10,00
58,10	737,00	24,57	1,00	33	264	62	1,62	11,30
44,40	1979,00	65,97	1,00	16	162	3	1,37	12,30
7,03	1190,00	39,67	1,00	41	547	2	1,20	9,90
3,40	1998,00	66,60	1,00	43	49	3	1,62	9,20
41,93	576,00	19,20	1,00	43	155	80	1,35	13,50
4,50	475,00	15,83	1,00	36	361	80	1,54	8,70
10,77	517,00	17,23	1,00	54	294	70	1,10	10,10
62,53	610,00	20,33	1,00	12	315	67	1,95	8,90
1,33	328,00	10,93	0,00	21	113	96	1,41	7,80
29,03	2026,00	67,53	0,00	52	232	2	1,78	10,40
56,53	2078,00	69,27	0,00	29	309	3	1,63	9,80
20,30	1985,00	66,17	0,00	35	187	3		9,60
4,73	2092,00	69,73	0,00	33	340	31	1,60	11,30
15,20	1372,00	45,73	0,00	57	315	115	1,21	11,70
0,37	1106,00	36,87	0,00	39	190	16	1,69	10,80
13,60	1549,00	51,63	0,00	42	135	6	1,17	12,10
15,07	1815,00	60,50	0,00	54	824	15	1,33	9,30
8,03	1234,00	41,13	0,00	34	204	4	2,94	9,00
14,90	1688,00	56,27	0,00	34	384	2	1,69	12,00
34,93	2033,00	67,77	0,00	30	394	4	3,03	9,50
12,27	1328,00	44,27	0,00	37	93	3	1,06	9,70
74,90	237,00	7,90	0,00	35	510	3	1,46	11,70
1,87	2077,00	69,23	0,00	34	237	4	1,17	11,40
98,30	2078,00	69,27	0,00	38	741	4	1,34	11,60

9,90	2078,00	69,27	0,00	41	224	5	1,38	10,60
23,17	2026,00	67,53	0,00	32	285	6	1,46	13,70
116,10	2012,00	67,07	0,00	29	352	4	1,24	12,80
58,67	321,00	10,70	0,00	38	703	3	1,52	7,60
1,53	328,00	10,93	0,00	3	0	4	1,21	14,40
20,40	1984,00	66,13	0,00	40	550	4	1,18	11,20
19,37	1985,00	66,17	0,00	42	261	2	1,36	11,80
10,07	1564,00	52,13	0,00	45	17	3	1,11	7,80
5,27	1274,00	42,47	0,00	54	324	2	1,29	9,90
84,13	2026,00	67,53	0,00	69	485	12	1,28	9,20
84,13	2026,00	67,53	0,00	54	495	5	1,35	12,10
19,60	1998,00	66,60	0,00	48	44	41	1,75	7,20
49,83	1991,00	66,37	0,00				3,31	10,40
-1,10	504,00	16,80	0,00	40	388	4	1,40	12,00
23,20	227,00	7,57	1,00	30	258	24	1,41	9,80
66,10	1978,00	65,93	0,00	17	340	6	1,41	11,00
14,97	2041,00	68,03	0,00	26	1031	2	1,19	11,90
22,90	1991,00	66,37	0,00	33	717	4	1,29	11,70
23,57	1992,00	66,40	0,00	31	209	1	1,45	13,10
24,80	1991,00	66,37	0,00				1,59	10,40
166,20	2092,00	69,73	0,00	38	628	10	1,65	10,90
43,17	2041,00	68,03	0,00				1,47	13,20
47,03	242,00	8,07	0,00	106	422	1	1,60	11,80
18,87	838,00	27,93	0,00	39	287	52	1,71	9,00
16,13	537,00	17,90	0,00	32	321	52	1,33	9,80
39,27	328,00	10,93	0,00	29	121	46	1,50	12,80
18,97	902,00	30,07	0,00	38	627	78	1,35	7,80
4,20	888,00	29,60	0,00	48	271	60	1,14	8,90
25,77	803,00	26,77	0,00	53	189	64	1,35	11,40
14,73	776,00	25,87	0,00	42	92	69	1,19	10,50
38,83	509,00	16,97	0,00	44	178	71	1,21	13,20
7,00	383,00	12,77	0,00	32	123	80	0,87	11,80
4,03	327,00	10,90	0,00	20	90	44	1,38	12,40
4,50	510,00	17,00	0,00	27	48	49	1,70	10,50
43,00	824,00	27,47	0,00	35	186	63	1,23	12,90
6,77	439,00	14,63	0,00	35	19	47	2,07	10,70
317,97	762,00	25,40	0,00	29	178	51	1,32	10,00
268,63	903,00	30,10	0,00	31	105	115	1,53	12,70
31,93	516,00	17,20	0,00	25	46	46	0,91	3,94
8,43	789,00	26,30	0,00	31	515	72	0,94	9,30
13,33	951,00	31,70	0,00	47	249	70	1,32	10,00
32,40	951,00	31,70	0,00	59	2	81	1,18	11,30
15,93	950,00	31,67	0,00	47	890	80	2,76	12,10
10,00	468,00	15,60	0,00	42	443	90	1,28	13,90
21,47	440,00	14,67	0,00	32	230	66	1,09	12,10
36,43	320,00	10,67	0,00	34	345	70	1,20	14,20
112,60	903,00	30,10	0,00	32	964	44	1,19	8,10
15,00	538,00	17,93	0,00	35	3	27	0,95	11,50
21,43	845,00	28,17	0,00	54	40	75	1,10	7,70
21,07	944,00	31,47	0,00	54	185	52	1,40	9,80

3,97	320,00	10,67	0,00	29	354	49	1,92	13,10
24,27	796,00	26,53	0,00	35	123	66	1,80	9,10
18,23	132,00	4,40	0,00	29	343	53	1,93	10,50
10,97	461,00	15,37	0,00	26	94	63	1,18	11,60
32,00	803,00	26,77	0,00	35	322	49	1,24	8,30
33,40	474,00	15,80	0,00	40	40	54	2,08	10,90
22,00	789,00	26,30	0,00	27	323	71	1,09	10,60
2,43	92,00	3,07	0,00	84	42	31	1,52	11,40
24,87	307,00	10,23	0,00	31	590	36	1,26	12,30
24,73	769,00	25,63	0,00	45	114	69	1,28	11,10
17,37	992,00	33,07	0,00	50	339	81	1,33	9,90
0,63	789,00	26,30	0,00	23	40	51	0,99	15,60
14,00	817,00	27,23	0,00	25	302	71	1,18	9,40
46,67	453,00	15,10	0,00	42	182	59	1,42	9,90
42,13	545,00	18,17	0,00	39	889	82	1,44	7,60
16,30	453,00	15,10	0,00	40	506	81	1,38	13,40
2,67	418,00	13,93	0,00	52	587	66	1,66	8,30
159,97	461,00	15,37	0,00	31	1327	87	1,31	13,10
3,67	377,00	12,57	0,00	18	657	73	1,83	12,20
44,07	517,00	17,23	0,00	40	762	85	1,26	11,70
2,97	384,00	12,80	0,00	28	173	47	0,88	9,70

HTC	albumin	Globulin	k	Na	P	Ca	LDL	HDL
32,20	3,10	2,70	5,10	133,00	4,70	8,00	131,00	32,00
30,80	4,00	3,70	6,40	141,00	4,30	9,70	#NULO!	15,00
33,40	3,50	3,80	4,90	139,00	3,70	8,90	97,00	41,00
30,30	3,70	2,90	6,10	139,00	5,40	8,90	54,00	35,00
32,50	4,00	3,10	6,00	138,00	5,20	9,40	67,00	30,00
35,30	4,00	3,50	5,60	137,00	3,20	9,20	115,00	40,00
37,20	3,80	2,60	5,40	141,00	3,90	9,10		
30,80	3,80	3,00	4,80	139,00	4,70	8,80	115,00	37,00
37,10	3,70	2,80	4,70	142,00	4,40	9,20	89,00	35,00
37,30	3,60	3,00	5,00	132,00	4,00	8,50	52,00	47,00
32,10	3,60	3,20	5,40	138,00	3,00	9,10	85,00	42,00
25,50	4,00	3,40	6,90	135,00	4,60	10,50	103,00	42,00
29,10	3,40	4,00	4,90	136,00	4,20	6,80	79,00	30,00
31,30	3,20	3,70	6,80	142,00	4,60	9,20	75,00	38,00
32,50	4,00	2,70	4,90	144,00	4,80	9,30	152,00	33,00
33,50	4,10	2,70	5,00	140,00	3,30	9,40		26,00
27,60	3,71	3,87	4,60	137,00	8,20	11,62	101,00	56,00
27,00	4,01	3,22	6,16	136,00	6,16	9,13	126,00	71,00
24,00	4,37	3,52	4,62	137,00	2,73	9,44	101,00	104,00
21,30	3,40	2,80	4,50	136,00	3,30	9,10	114,00	42,00
16,90	3,20	4,70	5,10	136,00	1,60	9,50	147,00	35,00
31,90	3,91	3,53	5,26	140,00	4,13	8,78	71,00	51,00
30,50	4,10	2,80	5,40	142,00	3,10	8,10	87,00	31,00
39,30	4,30	3,60	5,73	135,00	5,00	8,70	95,00	40,00
30,80	4,00	2,80	6,18	138,00	4,50	8,40	89,00	25,00
34,90	3,70	3,50	6,06	138,00	4,70	8,50	43,00	40,00
36,20	3,00	2,60	4,30	138,00	3,30	8,30	125,00	29,00
34,40	3,40	3,60	4,90	145,00	2,40	8,50	73,00	36,00
28,30	4,10	3,00	6,80	141,00	2,90	9,10		
40,40	3,80	2,60	5,47	135,00	4,70	8,50	89,00	35,00
27,90	3,90	3,10	5,40	140,00	8,40	9,10	100,00	100,00
30,90	3,60	4,00	5,67	140,00	4,00	8,50	60,00	61,00
26,10	4,10	3,00	5,00	131,00	5,70	8,10	122,00	48,00
22,80	4,34	3,93	5,14	141,00	5,16	12,80	78,00	45,00
37,50	3,80	2,80	5,50	137,00	5,00	9,00	92,00	33,00
35,00	4,10	2,80	5,10	137,00	5,50	9,00	87,00	54,00
36,80	4,30	2,90	4,50	139,00	6,00	9,60		17,00
28,70	3,90	2,80	4,70	140,00	4,20	9,40	133,00	31,00
36,90	4,20	2,80	5,00	140,00	4,10	9,10	80,00	26,00
34,00	4,70	3,20	6,10	136,00	4,70	6,90		
34,50	3,50	3,70	4,50	138,00	4,50	9,20	53,00	23,00
36,90	3,50	3,50	5,30	137,00	4,40	9,50	66,00	37,00
29,00	3,30	3,00	4,00	137,00	3,50	8,60	117,00	38,00
24,70	4,10	2,60	6,70	138,00	3,60	10,90	127,00	38,00
41,10	3,90	2,90	4,70	137,00	7,90	9,00	88,00	32,00
33,50	3,80	3,10	5,20	139,00	4,30	9,40	76,00	33,00
38,50	4,30	3,00	5,40	137,00	6,00	9,10	77,00	29,00
33,20	4,00	2,70	6,40	135,00	6,10	8,70		
26,80	3,70	3,40	5,40	141,00	6,50	9,10	83,00	28,00

30,50	3,70	2,80	4,20	143,00	2,50	8,50	63,00	31,00
39,70	4,30	3,40	5,70	141,00	7,90	9,90	108,00	34,00
36,30	4,10	2,80	4,80	138,00	4,80	9,20	106,00	26,00
27,40	4,20	2,90	5,70	141,00	5,10	9,40	124,00	47,00
44,10	4,10	2,80	5,80	142,00	8,40	8,30		
36,00	3,90	3,00	5,10	140,00	4,60	9,10	61,00	21,00
31,20	3,70	2,70	6,20	137,00	3,60	9,00	38,00	18,00
31,10	3,50	3,00	5,10	140,00	3,30	10,00	63,00	26,00
21,30	4,10	2,30	5,60	139,00	4,60	9,90	82,00	27,00
24,30	4,00	2,60	5,50	139,00	3,70	8,90	95,00	34,00
34,90	3,70	3,00	5,30	136,00	3,70	9,40	82,00	28,00
26,60	3,70	3,20	5,20	140,00	4,90	9,20	114,00	42,00
26,60	3,60	3,40	5,40	141,00	4,80	6,60	92,00	36,00
31,80	3,60	2,40	5,80	139,00	4,40	9,40	58,00	26,00
39,80	4,10	3,80	4,20	137,00	3,70	8,80	136,00	28,00
32,50	4,10	3,60	6,60	139,00	4,30	10,60		25,00
38,70	3,80	3,30	5,80	137,00	5,50	8,10	88,00	24,00
29,90	3,60	3,20	3,70	139,00	4,30	8,70	74,00	40,00
40,40	3,50	3,60	5,80	143,00	7,30	8,60	120,00	45,00
31,30	4,10	3,50	4,90	137,00	4,10	8,30	38,00	39,00
37,20	3,90	3,20	5,10	136,00	5,00	10,10	99,00	31,00
30,70	4,10	4,00	5,20	136,00	4,30	9,80	124,00	24,00
42,10	3,60	2,50	5,20	140,00	3,90	9,00	86,00	27,00
27,40	3,50	3,40	5,35	138,00	4,50	7,40	78,00	68,00
32,60	3,60	3,70	4,62	137,00	6,17	8,80	80,00	55,00
38,30	4,79	3,22	5,10	142,00	5,11	8,90	94,00	54,00
38,30	3,90	2,60	5,62	145,00	5,41	8,30	98,00	27,00
27,10	4,40	3,10	4,74	141,00	6,30	8,70	185,00	39,00
34,50	3,80	3,10	5,29	137,00	5,10	8,20	78,00	39,00
32,50	4,40	2,50	6,83	139,00	4,20	10,10	70,00	33,00
40,10	4,20	2,90	5,74	137,00	7,13	9,46	63,00	33,00
36,10	4,50	3,00	4,40	144,00	2,50	9,50	104,00	38,00
28,60	3,90	2,70	5,20	139,00	3,00	8,60	81,00	40,00
31,80	3,39	3,42	5,99	138,00	4,71	11,00	59,00	29,00
37,90	4,00	4,30	6,00	136,00	4,70	8,50	111,00	43,00
33,50	3,80	3,90	6,60	139,00	6,50	8,80	64,00	35,00
30,60	3,70	4,40	5,61	139,00	5,80	9,20	64,00	47,00
38,30	4,10	3,00	5,58	138,00	5,50	8,70	55,00	25,00
37,60	3,70	3,10	5,54	135,00	2,77	10,40	79,00	33,00
30,20	3,90	3,30	4,90	139,00	9,90	8,80	101,00	25,00
30,10	4,40	2,90	5,07	139,00	5,60	8,60	128,00	39,00
33,50	4,10	2,40	5,37	140,00	6,40	8,90	60,00	27,00
35,40	4,40	2,90	4,85	138,00	5,60	8,40	81,00	36,00
42,20	4,50	2,50	5,80	139,00	5,10	8,10	87,00	33,00
38,60	4,30	3,10	6,00	141,00	4,20	8,70	81,00	48,00
44,40	4,10	2,90	7,05	135,00	5,50	8,70	70,00	35,00
24,70	3,45	3,75	5,60	141,00	7,41	9,56	82,00	46,00
36,20	3,60	3,40	4,40	141,00	3,60	8,50	64,00	26,00
23,00	4,30	3,90	6,08	137,00	4,60	8,70	96,00	65,00
29,70	3,90	2,50	5,49	139,00	4,30	8,30	51,00	44,00

37,90	4,20	3,10	6,00	134,00	5,70	8,40	73,00	49,00
28,50	3,30	3,50	4,43	138,00	3,20	8,00	58,00	43,00
30,70	4,00	3,00	5,79	129,00	2,60	8,00	101,00	37,00
34,80	4,00	3,20	5,40	138,00	4,70	8,50	101,00	43,00
26,40	3,90	4,10	5,67	137,00	4,20	9,60	98,00	93,00
31,80	3,71	4,28	5,70	138,00	2,80	9,73	97,00	30,00
33,30	3,50	2,60	5,39	140,00	5,30	8,10	59,00	32,00
34,90	4,50	2,80	4,82	138,00	4,30	9,10	35,00	70,00
35,20	3,70	3,30	4,30	134,00	4,70	8,20	93,00	28,00
34,10	4,00	4,00	4,81	141,00	3,00	9,10	86,00	28,00
30,30	4,10	2,90	6,30	136,00	5,50	9,30	94,00	30,00
50,00	3,00	2,60	5,60	133,00	5,90	7,90	204,00	35,00
29,40	3,60	3,20	5,33	136,00	7,40	8,60	121,00	37,00
29,10	4,30	2,70	4,90	139,00	3,00	8,90	93,00	39,00
23,10	3,80	2,30	7,15	138,00	4,50	7,50	66,00	34,00
41,90	4,10	3,10	6,20	138,00	3,20	8,50	116,00	31,00
25,60	4,10	2,80	6,60	135,00	8,60	8,00	31,00	63,00
38,10	4,20	2,40	7,20	138,00	7,70	8,90	95,00	49,00
36,00	4,24	3,76	3,71	140,00	4,89	9,96	57,00	48,00
35,30	4,10	2,60	5,50	142,00	8,10	9,00	68,00	38,00
29,70	4,72	3,56	3,94	141,00	5,84	7,54		68,00

TG	Ferritin	Iron	Alkaline_phosphatase
110,00	234,00	66,00	62,00
499,00	475,00	58,00	99,00
134,00	287,00	143,00	247,00
168,00	497,00	53,00	119,00
151,00	65,00	61,00	229,00
64,00	231,00	47,00	365,00
	263,00	48,00	154,00
95,00	51,00	83,00	72,00
56,00	512,00	53,00	177,00
83,00	71,00	35,00	81,00
115,00	1068,00	56,00	395,00
183,00	524,00	43,00	135,00
179,00	166,00	41,00	100,00
120,00	790,00	47,00	119,00
332,00	425,00	53,00	337,00
731,00	284,00	60,00	188,00
173,00	30,51	56,00	73,00
51,00	110,00		202,00
55,00	544,00	20,00	103,00
112,00	887,00	17,00	117,00
160,00	504,00	29,00	134,00
73,00	140,50		107,00
234,00	476,00	69,00	111,00
107,00	539,30	85,00	77,00
422,00	84,00	67,00	1210,00
275,00	420,00	32,00	98,00
183,00	316,00	70,00	82,00
85,00		55,00	69,00
	1422,00	49,00	67,00
157,00	23,00	63,00	137,00
73,00	60,00	29,00	65,00
74,00	462,00	46,00	104,00
177,00	382,00	75,00	175,00
318,00	318,00		197,00
49,00	215,00	60,00	103,00
84,00	497,00	80,00	71,00
714,00	411,00	41,00	73,00
372,00	1749,00	46,00	67,00
112,00	202,00	56,00	41,00
	100,00	49,00	110,00
178,00	152,00	27,00	180,00
91,00	290,00	43,00	75,00
119,00	210,00	51,00	92,00
159,00	683,00	52,00	121,00
68,00	94,00	38,00	200,00
118,00	315,00	95,00	101,00
72,00	714,00	50,00	299,00
	61,00	80,00	92,00
147,00	561,00	58,00	83,00

73,00	117,00	54,00	161,00
78,00	574,00	52,00	85,00
161,00	317,00	49,00	257,00
77,00	1183,00	104,00	81,00
	71,00	96,00	61,00
163,00	168,00	82,00	63,00
123,00	422,00	114,00	88,00
83,00	415,00	49,00	170,00
66,00	475,00	85,00	93,00
112,00	525,00	101,00	182,00
63,00	714,00	44,00	130,00
101,00	1314,00	31,00	125,00
55,00	279,00	47,00	160,00
50,00	512,00	54,00	93,00
199,00	307,00	60,00	92,00
636,00	801,00	64,00	82,00
117,00	313,00	47,00	229,00
92,00	553,00	65,00	162,00
52,00	209,00	37,00	55,00
57,00	266,00	36,00	72,00
83,00	361,00	45,00	111,00
241,00	237,00	46,00	291,00
44,00	475,00	104,00	54,00
49,00	76,00	32,00	163,00
79,00	443,00	62,00	170,00
78,00	75,22	80,00	124,00
237,00	25,03	41,00	448,00
99,00	270,00	61,00	57,00
114,00	140,00	36,00	62,00
150,00	111,00	32,00	111,00
46,00	140,00	61,00	270,00
462,00	435,00	45,00	74,00
121,00	249,00	115,00	95,00
125,00	1314,00	33,00	157,00
117,00	196,00	67,00	53,00
115,00	716,00	50,00	62,00
122,00	164,00	81,00	296,00
106,00	39,00	58,00	81,00
161,00	514,00	81,00	390,00
197,00	116,00	50,00	48,00
88,00	198,00	58,00	74,00
126,00	93,00	102,00	90,00
181,00	54,00	97,00	69,00
333,00	107,80	68,00	59,00
170,00	87,90	46,00	120,00
81,00	57,00	29,00	100,00
190,00	218,00	57,00	422,00
336,00	263,00	58,00	57,00
115,00	31,00	63,00	40,00
81,00	46,00	62,00	70,00

214,00	305,00	34,00	73,00
131,00	56,00	36,00	119,00
380,00	108,00	66,00	138,00
170,00	270,00	70,00	74,00
77,00	46,00	26,00	145,00
88,00	576,00	39,00	253,00
216,00	137,00	73,00	73,00
86,00	388,00	55,00	97,00
114,00	185,00	47,00	245,00
325,00	43,70	70,00	39,00
129,00	196,00	24,00	56,00
341,00	638,00	111,00	83,00
150,00	56,00	38,00	82,00
86,00	69,10	38,00	114,00
62,00	632,00	22,00	578,00
344,00	18,20	53,00	186,00
255,00	3,40	34,00	83,00
237,00	152,00	46,00	153,00
151,00	102,30	91,00	318,00
185,00	272,60	52,00	131,00
502,00	114,70		121,00
