# Supplementary material for: The prognostic value of gait speed in hemodialysis patients: A prospective observational study
Source: PLoS One. 2026 Mar 18;21(3):e0343612. doi: 10.1371/journal.pone.0343612 (PMC12998855; doi:10.1371/journal.pone.0343612)
Supplement: Attachment — Submitted filename: REVIEWER_COMMENTS_.docx [file pone.0343612.s004.docx]

[Emily Chenette](https://www.google.com/search?q=Emily+Chenette&oq=PLOS+ONE+EDITOR+IN+CHIEF&gs_lcrp=EgZjaHJvbWUqDAgAEAAYExjjAhiABDIMCAAQABgTGOMCGIAEMgkIARAuGBMYgAQyCggCEAAYExgWGB4yCggDEAAYExgWGB4yCggEEAAYExgWGB4yBwgFEAAY7wUyCggGEAAYgAQYogQyCggHEAAYgAQYogTSAQg1NDcyajBqN6gCCLACAfEFzzK38qiyx7U&sourceid=chrome&ie=UTF-8&mstk=AUtExfAXR5Jm0049mHXQM8PcYlGfIaikJr5D6Eh-UxlNz4IrmVe1bNbUDHZrRdl0MsowYJU7ZZLeaBgWMccaJm1Ui6mhYhTTL16hoROv8n37nsG0mqabsA_uaR4Dz5FzgCJAJAjuTfTNDcfgcox6Id-9UymgjuJV0UMxeSA5yAo7C-QCOTI&csui=3&ved=2ahUKEwi9v9n91KGRAxWRCrkGHQU_PccQgK4QegQIAhAE), PhD
Editor
PLOS ONE

Manuscript Number: **PONE-D-25-31233**

Dear Editor,

I am attaching the revised version of our paper titled "The prognostic value of gait speed in hemodialysis patients: A prospective observational study” for consideration as an article in PLOS ONE. The revised sections are highlighted in yellow to emphasize the changes made.

We want to express our gratitude for the opportunity to review our document and make improvements to key points, thereby meeting the journal's requirements and addressing the reviewers’ suggestions. Formatting and quality have been adjusted in accordance with the guidelines provided in the author's guide. We have implemented all the suggested changes, and the following pages contain our point-by-point responses to the queries raised by the reviewers. We have addressed the proposed notes and provided clarification for any remaining questions.

With these revisions and responses, we believe that our work is now ready for acceptance by this esteemed journal.

Yours sincerely,

Vanessa Amaral Mendonça

The corresponding author*

**REVIEWER COMMENTS:**

Journal Requirements:

When submitting your revision, we need you to address these additional requirements.

1. Please ensure that your manuscript meets PLOS ONE's style requirements, including those for file naming. The PLOS ONE style templates can be found at

<https://journals.plos.org/plosone/s/file?id=wjVg/PLOSOne_formatting_sample_main_body.pdf> and

[https://journals.plos.org/plosone/s/file?id=ba62/PLOSOne_formatting_sample_title_authors_affiliations.pdf](https://track.editorialmanager.com/CL0/https:%2F%2Fjournals.plos.org%2Fplosone%2Fs%2Ffile%3Fid=ba62%2FPLOSOne_formatting_sample_title_authors_affiliations.pdf/1/010f019ab47f050d-c737d6c0-9936-494b-8b90-ef3268422dea-000000/i_iDiVi2e1Q3AomEhc5vYqgCubSnNKLkKheUI1b5kFw=238)

**Answer:** Thank you. Manuscript meets PLOS ONE's style requirements, including those for file naming.

2. Thank you for stating the following financial disclosure:
“The author(s) received no specific funding for this work.”

At this time, please address the following queries:

a) Please clarify the sources of funding (financial or material support) for your study. List the grants or organizations that supported your study, including funding received from your institution.

**Answer:** This work didn’t receive funding from my institution.

b) State what role the funders took in the study. If the funders had no role in your study, please state: “The funders had no role in study design, data collection and analysis, decision to publish, or preparation of the manuscript.”

**Answer:** The funders had no role in study design, data collection and analysis, decision to publish, or preparation of the manuscript.

c) If any authors received a salary from any of your funders, please state which authors and which funders.

**Answer:** The authors didn’t receive any salary from any of your funders.

d) If you did not receive any funding for this study, please state: “The authors received no specific funding for this work.”

**Answer:** This work didn’t receive any specific funding, but we must acknowledge some Brazilian agency research support. This is crucial for maintaining our line of research in Brazil. It’s essential to receive grants in our country.

“The authors thank the Coordenação de Aperfeiçoamento de Pessoal de Nível Superior (CAPES), Brazil; Conselho Nacional de Desenvolvimento Científico e Tecnológico (CNPq), Brazil; Fundação de Amparo à Pesquisa do Estado de Minas Gerais (FAPEMIG) (APQ-00709-24; APQ-01522-23), Brazil; and Hemodialysis Center of Santa Casa de Caridade de Diamantina, Minas Gerais, Brazil.”

Please include your amended statements within your cover letter; we will change the online submission form on your behalf.

3. Thank you for stating the following in the Acknowledgments Section of your manuscript:
“The authors thank the Coordenação de Aperfeiçoamento de Pessoal de Nível Superior (CAPES), Brazil; Conselho Nacional de Desenvolvimento Científico e Tecnológico (CNPq), Brazil; Fundação de Amparo à Pesquisa do Estado de Minas Gerais (FAPEMIG) (APQ-00709-24; APQ-01522-23), Brazil; and Hemodialysis Center of Santa Casa de Caridade de Diamantina, Minas Gerais, Brazil.”

We note that you have provided funding information that is not currently declared in your Funding Statement. However, funding information should not appear in the Acknowledgments section or other areas of your manuscript. We will only publish funding information present in the Funding Statement section of the online submission form.
Please remove any funding-related text from the manuscript and let us know how you would like to update your Funding Statement. Currently, your Funding Statement reads as follows:
“The author(s) received no specific funding for this work.”

Please include your amended statements within your cover letter; we will change the online submission form on your behalf.

4. We note that your Data Availability Statement is currently as follows: All relevant data are within the manuscript and its Supporting Information files.

Please confirm at this time whether or not your submission contains all raw data required to replicate the results of your study. Authors must share the “minimal data set” for their submission. PLOS defines the minimal data set to consist of the data required to replicate all study findings reported in the article, as well as related metadata and methods (<https://journals.plos.org/plosone/s/data-availability#loc-minimal-data-set-definition>).

For example, authors should submit the following data:

- The values behind the means, standard deviations and other measures reported;
- The values used to build graphs;
- The points extracted from images for analysis.

Authors do not need to submit their entire data set if only a portion of the data was used in the reported study.

If your submission does not contain these data, please either upload them as Supporting Information files or deposit them to a stable, public repository and provide us with the relevant URLs, DOIs, or accession numbers. For a list of recommended repositories, please see <https://journals.plos.org/plosone/s/recommended-repositories>.

If there are ethical or legal restrictions on sharing a de-identified data set, please explain them in detail (e.g., data contain potentially sensitive information, data are owned by a third-party organization, etc.) and who has imposed them (e.g., an ethics committee). Please also provide contact information for a data access committee, ethics committee, or other institutional body to which data requests may be sent. If data are owned by a third party, please indicate how others may request data access.

**Answer:** We confirm that all raw data required to replicate the results of this study are provided. The data are available in a PDF file included as Supplementary Material. This file contains all data used in the analyses reported in the manuscript.

5. If the reviewer comments include a recommendation to cite specific previously published works, please review and evaluate these publications to determine whether they are relevant and should be cited. There is no requirement to cite these works unless the editor has indicated otherwise. 
[Note: HTML markup is below. Please do not edit.]
**Answer:** Thank you. The recommendation to cite specific previously published works was reviewed and evaluated.

**REVIEWERS' COMMENTS:**

Reviewer's Responses to Questions

**Comments to the Author**

1. Is the manuscript technically sound, and do the data support the conclusions?

The manuscript must describe a technically sound piece of scientific research with data that supports the conclusions. Experiments must have been conducted rigorously, with appropriate controls, replication, and sample sizes. The conclusions must be drawn appropriately based on the data presented.

Reviewer #1: Yes

Reviewer #2: Partly

Reviewer #3: Yes

2. Has the statistical analysis been performed appropriately and rigorously?

Reviewer #1: Yes

Reviewer #2: No

Reviewer #3: Yes

3. Have the authors made all data underlying the findings in their manuscript fully available?

The [PLOS Data policy](http://track.editorialmanager.com/CL0/http:%2F%2Fwww.plosone.org%2Fstatic%2Fpolicies.action%23sharing/1/010f019ab47f050d-c737d6c0-9936-494b-8b90-ef3268422dea-000000/KHu6uZ8PO7P4koa3MpjwP-8IdXujfmA-UaW1cs9AEmI=238) requires authors to make all data underlying the findings described in their manuscript fully available without restriction, with rare exception (please refer to the Data Availability Statement in the manuscript PDF file). The data should be provided as part of the manuscript or its supporting information, or deposited to a public repository. For example, in addition to summary statistics, the data points behind means, medians and variance measures should be available. If there are restrictions on publicly sharing data—e.g. participant privacy or use of data from a third party—those must be specified.

Reviewer #1: Yes

Reviewer #2: No

Reviewer #3: No

4. Is the manuscript presented in an intelligible fashion and written in standard English?

PLOS ONE does not copyedit accepted manuscripts, so the language in submitted articles must be clear, correct, and unambiguous. Any typographical or grammatical errors should be corrected at revision, so please note any specific errors here.

Reviewer #1: Yes

Reviewer #2: Yes

Reviewer #3: Yes

5. Review Comments to the Author

Please use the space provided to explain your answers to the questions above. You may also include additional comments for the author, including concerns about dual publication, research ethics, or publication ethics. (Please upload your review as an attachment if it exceeds 20,000 characters)

**Reviewer #1:** Thank you for the invitation to review this paper, Dr. Bataglia, below are my comments

- Could the authors clarify whether the gait speed cutoff of 1.13 m/s was derived exclusively from their ROC analysis, or if it was also compared to previously established thresholds in similar populations?

**Answer:** Thank you for this comment. The gait speed cutoff of 1.13 m/s was derived from the ROC curve analysis performed in our sample, aiming to identify the threshold with the best balance between sensitivity and specificity for the studied outcome. Subsequently, we conducted a comparative analysis with gait speed cutoffs reported in previous studies involving populations with chronic diseases to assess the external validity of our findings. This comparison showed that the identified cutoff is slightly higher, which can be attributed to the younger age range of the population included in this study. This question is explored in the Discussion section (page 12, lines 247-253).

- In the sample size calculation, the authors mention an expected event rate of 34%. Could they explain how this percentage was determined (e.g., from prior literature or preliminary data)?

**Answer:** Thank you for this comment. An initial literature review was conducted to identify mortality rates reported in similar populations. Subsequently, a pilot study was performed, and the observed mortality rate of 34% was found to be consistent with values previously described in the literature.

**References:**

1. de Jager DJ, Grootendorst DC, Jager KJ, et al. Cardiovascular and Noncardiovascular Mortality Among Patients Starting Dialysis. *JAMA.* 2009;302(16):1782–1789. doi:10.1001/jama.2009.1488

2. Barra, A.B.L., Roque-da-Silva, A.P., Canziani, M.E.F. *et al.* Characteristics and predictors of mortality on haemodialysis in Brazil: a cohort of 5,081 incident patients. *BMC Nephrol* **23**, 77 (2022). https://doi.org/10.1186/s12882-022-02705-x

- The results indicate that phosphorus was associated with mortality in univariate analysis but not in the adjusted model. Could the authors elaborate on possible reasons for this attenuation?

**Answer:** Thank you for this comment. Cox proportional hazards regression is a time-to-event analysis that estimates the association between covariates and the risk of the studied outcome, allowing the identification of independent predictors when multiple variables are included in the model. In the univariate analysis, serum phosphorus was significantly associated with mortality when evaluated in isolation. However, this association was attenuated in the multivariate model after adjustment for functional performance. This is a mathematical attenuation, and it likely occurred because, when analyzed together, functional performance captures a broader and more integrated representation of the patients' clinical and physiological status, thereby overlapping the prognostic information provided by serum phosphorus alone.

- Also, the paper didn't the criteria for an acceptable ROC-AUC value, consider this literature to help clarify an acceptable threshold <https://doi.org/10.1080/23249935.2025.2516817> (typically 0.8 and above).

**Answer:** Thank you for this comment. In this study, an AUC greater than 0.7 was considered acceptable, while an AUC greater than 0.8 was considered excellent for the proposed cutoffs, based on the literature (Hajian-Tilaki, 2013). The AUC to identify mortality hazards in hemodialysis patients using gait speed was 0.85 (0.77-0.90), p < 0.0001, indicating an excellent result. We thank the reviewer for the suggestion. The recommended reference has been incorporated into the Methods and References sections (Reference 30).

**Reference:** Hajian-Tilaki K. Receiver Operating Characteristic (ROC) Curve Analysis for Medical Diagnostic Test Evaluation. Caspian J Intern Med. 2013;4(2):627–35.

- In the causes of death reported, some were listed as “undetermined origin.” Could the authors clarify how these cases were classified and whether they might influence the robustness of the survival analysis?

**Answer:** Thank you for this comment. Deaths classified as “undetermined origin” occurred in cases in which the patient died within their municipality and detailed information on the cause of death was not available in the medical records or official registries at the time of data collection. Importantly, these cases were classified as death events in the survival analysis regardless of the specific cause. As the primary outcome of this study was all-cause mortality, the absence of cause-specific information does not compromise the robustness of the Cox regression models. In addition, deaths due to external causes, such as accidents, trauma, or other non-disease-related events, were neither reported nor observed in this cohort.

- The manuscript highlights gait speed as a “free-of-charge measure.” Could the authors expand briefly on the feasibility of implementing this in dialysis centers with limited staffing or high patient loads?

**Answer:** Integrating the gait speed test into routine dialysis assessments is a practical and cost-effective strategy to enhance patient monitoring, making it particularly suitable for dialysis centers with limited staffing or high patient volumes. The test requires minimal and straightforward training, as the procedure involves only a standardized environment (an 8-meter corridor) and simple instructions (Cruz-Jentoft et al., 2019). It is quick to administer and can be scheduled at regular intervals, such as monthly or quarterly, without interfering with the dialysis routine. This integration enables longitudinal follow-up of functional capacity, providing prognostic and clinically relevant information with minimal burden on healthcare resources.

**Reference:** Cruz-Jentoft AJ, Bahat G, Bauer J, Boirie Y, Bruyère O, Cederholm T, et al. Sarcopenia: revised European consensus on definition and diagnosis. Age Ageing. 2019 Jan 1;48(1):16–31.

- The generalizability of findings is noted as a limitation, given the single-center design. Could the authors provide more detail on the characteristics of this center (e.g., patient demographics, resources) to help readers assess comparability with other settings?

**Answer:** Thank you for highlighting this important point regarding generalizability. The study was conducted in a single outpatient hemodialysis center located in a region with a low Human Development Index (HDI), which influences both patient characteristics and healthcare delivery. The center provides chronic hemodialysis predominantly within a public healthcare system, and the patient population is characterized by a wide age range, with a predominance of younger adults compared to cohorts commonly reported in high-income settings. This demographic profile reflects regional epidemiological patterns, including earlier onset of chronic kidney disease and differences in access to preventive care. Additionally, the center operates with standard staffing levels and routinely available resources, without access to specialized rehabilitation or infrastructure, which is representative of many dialysis units in low- and middle-income regions. The information was clarified in the discussion.

**Modified text:** Page 13, lines 269-279.

**Reviewer #2:**

1. Phosphorus (HR 0.72 [95% CI 0.54–0.96], p = 0.03). Can I interpret this as: For each unit increase in serum phosphorus, the risk of HD-related mortality decreases by 18%? This does not align with clinical understanding.

**Answer:**

The hazard ratio below 1 observed for serum phosphorus in the univariate Cox regression (HR 0.72 [95% CI 0.54–0.96]) reflects a statistical association within the range of values observed in our sample. It should not be interpreted as a general protective effect of higher phosphorus levels. Importantly, Cox regression assumes a linear relationship between the covariate and the log hazard, which may oversimplify potentially non-linear or threshold-based biological associations.

Although serum phosphorus levels differed between the survival and non-survival groups, both groups presented mean values within a relatively preserved range (survival: 5.04 ± 1.51 mg/dL; non-survival: 4.31 ± 1.40 mg/dL). Hyperphosphatemia is a defined prognostic factor in hemodialysis patients, however previous studies indicate that increased mortality risk is particularly associated with low phosphorus levels (e.g., < 3 mg/dL) (Lertdumrongluk et al., 2013; Lee et al., 2017), often reflecting malnutrition or protein-energy wasting. In this context, lower phosphorus concentrations in our sample may act as a surrogate marker of poorer nutritional and functional status rather than indicating a direct causal relationship.

Accordingly, despite the univariate association, serum phosphorus did not remain significant in the multivariate model after adjustment for functional capacity, suggesting that its prognostic effect is attenuated when more integrative clinical variables are considered.

This contextualization was added in the Discussion section.

**Modified text:** Page 11, lines 217-226.

**References**

1. Lertdumrongluk P, Rhee CM, Park J, Lau WL, Moradi H, Jing J, Molnar MZ, Brunelli SM, Nissenson AR, Kovesdy CP, Kalantar-Zadeh K. Association of serum phosphorus concentration with mortality in elderly and nonelderly hemodialysis patients. J Ren Nutr. 2013 Nov;23(6):411-21. doi: 10.1053/j.jrn.2013.01.018. Epub 2013 Apr 28. PMID: 23631888; PMCID: PMC3735629.

2. Lee J-E, Lim J-H, Jang HM, Kim YS, Kang S-W, Yang CW, et al. (2017) Low serum phosphate as na independent predictor of increased infection- related mortality in dialysis patients: A prospective multicenter cohort study. PLoS ONE 12(10): e0185853. <https://doi.org/10.1371/journal>. pone.0185853

2. HR = 0.04 [95% CI 0.00–0.14], approaching zero, which similarly defies logic.

**Answer:** The hazard ratio indicates a strong protective association between gait speed and hemodialysis-related mortality. The HR = 0.04 (95% CI 0.00–0.14) is interpreted as “For each unit increased in gait speed, the risk of HD-related mortality decreases by 96%)”. A typographical error in the Abstract regarding the confidence interval has been corrected from *HR = 0.04 [95% CI 0.00–0.14]* to *HR 0.04 (95% CI 0.01–0.15)*, with no impact on the interpretation of the results.

**Modified text:** Page 2, lines 40-41.

3. I don't understand what Table 2 is trying to convey. What does “Low mobility” refer to? And which covariates were adjusted for?

**Answer:** Low mobility was defined as a gait speed < 1.13 m/s, based on the cutoff value determined by ROC analysis. The adjusted model included sex, age, body mass index, hemodialysis vintage, presence of diabetes, hemoglobin level, phosphorus, and albumin level. These specifications are presented in the legend of Table 2.

**Reviewer #3:**

The submitted manuscript presents an extremely relevant topic in the clinical context of dialysis, presenting a variable that is easy to measure and low cost. The article has strengths regarding the sample size, analysis time, and robust statistical treatment. It is suggested that the contact information for sample recruitment be included. Considering the additional analyses of gait speed, even though an adjusted regression analysis was performed, it is considered relevant to discuss the influence of variables such as age, body composition, and significant biochemical parameters on gait speed and mobility. Although dialysis time and none of the aspects of body composition showed statistical significance, citing and discussing the possible influences on gait is fundamental (Discussion Topic).

Below are the requests for technical adjustments to the text:
• It seems that the phosphorus and albumin values are swapped in the table, as indicated in the article's discussion;

**Answer:** Thank you for this observation. The values presented in the table have been verified and are correct. The text in the Discussion was corrected from “had higher levels of albumin and phosphorus” to “had higher levels of albumin and lower levels of phosphorus”.

**Modified text:** Page 11, lines 216-217.

• I believe it would be interesting to briefly explain the methods for biochemical analyses;

**Answer:** Thank you for this comment. The biochemical analyses were performed by the laboratory’s internal sector, and the techniques used were briefly described in the table. This information was added in the supplementary material (S1) and cited in the Methods section.

**Modified text:** Page 5, lines 115-117.

| **Biochemist Variables** | **Method** |
| --- | --- |
| Vit D (ng/ml) | Chemiluminescence |
| PTH (pg/ml) | Chemiluminescence |
| C-PR (pg/ml) | Turbidimetry |
| Kt/v | Urea Kinetic Modeling |
| Hemoglobin (g/dL) | Automated counting by flow cytometry |
| Hematocrit (%) | Automated counting by flow cytometry |
| Albumin (g/dL) | Colorimetric |
| Globulin (g/dL) | Immunonephelometry |
| Potassium (g/dL) | Ion-selective electrode |
| Sodium (g/dL) | Ion-selective electrode |
| Phosphorus (g/dL) | Colorimetric |
| Calcium (mg/dL) | Colorimetric |
| LDL (mg/dL) | Enzymatic colorimetric |
| HDL (mg/dL) | Enzymatic colorimetric |
| Triglycerides (mg/dL) | Enzymatic colorimetric |
| Ferritin (ng/dL) | Chemiluminescence |
| Iron (μg/dL) | Colorimetric |
| Alkaline phosphatase (U/L) | Colorimetric |

• Adjust the table legends regarding the abbreviations.

**Answer:** Thank you for this comment. The table legends were revised to ensure clarity of the abbreviations.

6. PLOS authors have the option to publish the peer review history of their article ([what does this mean?](https://track.editorialmanager.com/CL0/https:%2F%2Fjournals.plos.org%2Fplosone%2Fs%2Feditorial-and-peer-review-process%23loc-peer-review-history/1/010f019ab47f050d-c737d6c0-9936-494b-8b90-ef3268422dea-000000/cXkUAhxoyAP_qjurfv_MF2Iks18Tg_XSrn71mJDSDhM=238)). If published, this will include your full peer review and any attached files.


If you choose “no”, your identity will remain anonymous but your review may still be made public.


**Do you want your identity to be public for this peer review?** For information about this choice, including consent withdrawal, please see our [Privacy Policy](https://track.editorialmanager.com/CL0/https:%2F%2Fwww.plos.org%2Fprivacy-policy/1/010f019ab47f050d-c737d6c0-9936-494b-8b90-ef3268422dea-000000/pH_2cVK8HPatUjtytdBqdPHAUisWN-P5QlnIHVLBE9M=238).

Reviewer #1: No

Reviewer #2: No

Reviewer #3: No
